# The case of galantamine: repurposing and late blooming of a cholinergic drug

**DOI:** 10.4155/fso.15.73

**Published:** 2015-09-03

**Authors:** Hermann AM Mucke

**Affiliations:** 1 H. M. Pharma Consultancy, Enenkelstrasse 28/32, A-1160 Wien (Vienna), Austria

**Keywords:** Alzheimer’s disease, cholinesterase inhibitors, drug repositioning, galantamine, intellectual property

## Abstract

Galantamine is a reversible inhibitor of cholinesterases and an allosteric modulator of neuronal nicotinic acetylcholine receptors which restores reduced cholinergic tone in the central and peripheral nervous system. Characterized in the early 1950s in Bulgaria, it saw limited use for paralytic and neuropathic conditions until the cholinergic hypothesis of Alzheimer’s disease opened totally new perspectives for its utility. Although constricted supplies at extremely high prices and a fragmented patent situation made its repurposing challenging, galantamine was globally launched as an Alzheimer’s disease drug in 2000. Many other possible uses have been clinically investigated, and might yet develop into another drug career. This case study is presented as an example for classical on-target drug repurposing and the challenges that such a project can face.

## Abstract (lay abstract)

Acetylcholine is a key neurotransmitter that regulates many functions in the CNS, as well as in other body organs, through the cholinergic system. Galantamine is a well-known alkaloid drug that modulates this system toward a higher cholinergic tone, and has been put to several therapeutic uses during the past decades, most recently, for Alzheimer’s disease. However, its known actions might allow repurposing toward even more applications not only in psychiatry and neurology but also in eye medicine and even in immunology. This article summarizes the claims, the evidence and the potential of galantamine beyond its currently approved uses.

**Figure 1.**
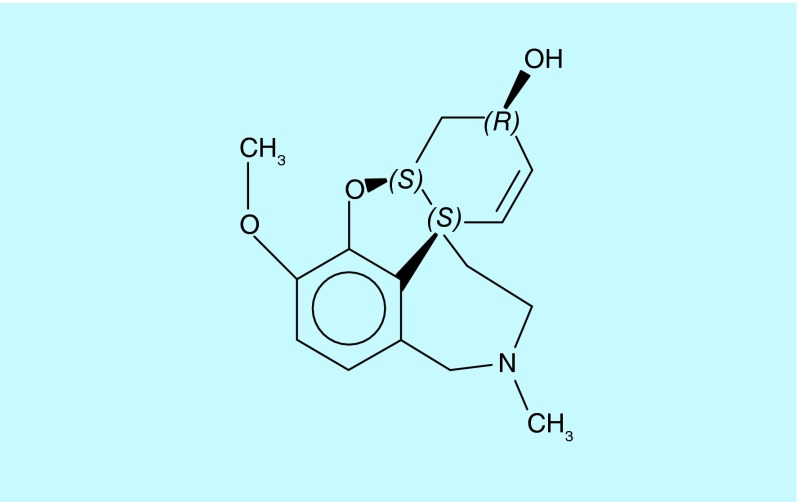
**Galantamine.**

## An almost obscure early history

In 1947, a Soviet journal reported the presence of previously unknown alkaloids in the common snowdrop, *Galanthus nivalis*; the authors named the major (yet undefined) compound galanthamine [1]. A few years later, the same team isolated and characterized it from the closely related *Galanthus woronowii* [2]. Japanese researchers appear to have isolated the same alkaloid from the red spider lilly (*Lycoris radiata*), calling it lycoremine [3]. It seems likely that ethnomedicine prompted these investigations [4], although hard evidence is lacking. Eventually, a quite complicated chemical structure with three chiral centers was revealed: galantamine is (4a*S*,6*R*,8a*S*)-5,6,9,10,11,12-hexahydro-3-methoxy-11-methyl-4aH-[1]benzofuro[3a,3,2-ef][2] benzazepin-6-ol (see Figure 1).

The discovery generated very limited international interest until 1960, when therapeutic usefulness was suggested based on the finding that the alkaloid is an inhibitor of cholinesterases, with stronger activity toward muscle acetylcholinesterase than pyridostigmine but somewhat less than neostigmine [5]. Almost immediately, galanthamine (the ‘h’ was dropped only in the 1990s when the international nonproprietary name was defined) was added to the armamentarium provided by these older cholinesterase inhibitors to treat myopathies or postpolio paralytic conditions, and for reversal of neuromuscular blockade after anesthesia [6,7]. It was also found useful for the treatment of peripheral neuropathies and radiculitis [8], where oral doses of 15–25 mg/day given for up to 80 days supplemented initial subcutaneous injections or iontophoretic infiltrations. The use of galantamine, usually under the Nivalin^®^ trade name, was mostly limited to Bulgaria, Italy, France and Germany during the 1960s through the 1980s.

While these are all peripheral actions, galantamine (a tertiary ammonium base) easily penetrates the blood–brain barrier and also inhibits brain cholinesterases, increasing central cholinergic tone [9]. This certainly contributed to the drug’s action in spinal poliomyelitis and awakening from anesthesia, and also to the positive effects reported in intracerebral hemorrhage from various causes [10]. There is anecdotal evidence that some European neuropsychiatrists used Nivalin off-label to treat cognitive and emotional impairments after traumatic brain injury. However, no case reports or studies were published.

In 1977, Baraka and Harik, perhaps following up on earlier Soviet Union animal data, reported that galantamine (0.5 mg/kg iv.) reversed the acute anticholinergic syndrome (drowsiness and disorientation up to delirium) that the muscarinic receptor antagonist, scopolamine induces in cognitively normal volunteers [11] but they made no attempt to quantify cognitive restitution in their subjects. With hindsight, we might be tempted to comment that they missed a great opportunity. But not only was psychiatry still struggling toward a firm concept of cholinergic involvement in cognition at this time (it was only in 1982 that the cholinergic hypothesis of geriatric memory dysfunction was advanced [12]) but there were also hardly any suitable tests available to measure acutely impaired cognitive performance.

## The takeoff that did not happen

However, once the cholinergic hypothesis of Alzheimer’s disease was accepted (which happened quickly because the lack of cholinergic tone in patients’ brains was demonstrated only a year later [13]), perspectives for an additional use of galantamine as an antidementive drug should have been immediately evident. While its blockade of enzymatic action is competitive with acetylcholine and totally reversible [14], its mean terminal half-life in plasma after oral administration is 5–6 h [15] and it has very few side effects beyond the typical cholinergic ones. These three crucial elements set it apart from other cholinesterase inhibitors available at this time, and should have made it much more suitable for development as a drug for Alzheimer’s disease.

But the fact that galantamine was available only from botanical sources, which have contents around 0.1%, with the only commercial suppliers for export (in Bulgaria) charging prices around US$40,000/kg and no industrially acceptable synthesis in sight (although laboratory-scale syntheses with yields in the single-digit percent range had been reported in the 1960s) made its development unattractive for the pharmaceutical industry. Tacrine (a simple aminoacridine with considerable hepatotoxicity) became the first drug to be specifically approved for treating cognitive symptoms of Alzheimer’s disease, followed by the chemically more complex donepezil, which however was still quite easy to synthesize.

## Full synthesis versus plant extraction

Evidently, the supply bottleneck had to be removed to enable development, which could ultimately require galantamine to be reliably available in the low metric ton scale on a yearly basis. This problem was solved during the 1990s using two different approaches that were conventional but effective: an extraction process was developed that used readily available daffodil bulbs; and – more importantly - the full chemical synthesis was upscaled and optimized, increasing yields by an order of magnitude and ultimately allowing batch sizes in the range of 100 kg to be manufactured under GMP-compliant conditions [16]. Regulatory clinical trials were conducted (for a summary see [17]), and in 2000 galantamine was launched in the USA and Europe for the symptomatic treatment of Alzheimer’s disease, originally as Reminyl^®^, which was later changed to Razadyne^®^ in the USA.

## A saga of corporate conflict & resolution

The later part of this chronology, which can be assembled from public resources easily enough, might create the impression that the development of galantamine as an Alzheimer drug, while belated and slow, was ultimately a straightforward and well-coordinated process. This is rather remote from the truth. The path from Nivalin to Reminyl, while not burdened by efficacy failures or safety problems in clinical studies, was tortuous for entirely different reasons. The root cause of these problems was the fragmentation of commercial and intellectual property rights.

The use of galantamine for Alzheimer’s disease was covered by the US patent no. 4,663,318 (priority date 1986) and its international equivalents, held by Synaptech, Inc. In the 1990s, this small US company teamed with Shire Pharmaceuticals of the UK, which at that time had a track record of inlicensing promising compounds for early-stage clinical development in order to outlicense the successful candidates for Phase III trials and marketing. In the case of galantamine, Shire’s partner was the Johnson and Johnson company, Janssen Pharmaceuticals.

In the meantime, a small private Austrian company, Waldheim Pharmazeutika had secured additional Austrian regulatory approval to market its Nivalin tablets for Alzheimer’s (the first such approval for galantamine) and promoted it accordingly, prompting Shire to commence patent infringement procedures. Waldheim, which had no use patents for galantamine, responded with invalidation lawsuits against the Synaptech patent, alleging non-enabling disclosure (the ‘318 patent did not cite any supporting clinical or animal data) and obviousness (based on the literature). In parallel, Waldheim proceeded with the optimization of its industrial-scale synthesis for galantamine, for which it had filed its first patent application in 1994 (later published as WO/1996/012692).

Eventually, it became obvious that any global-scale galantamine project that did not productively involve all concerned parties would either have to exclude interesting markets or else face high risks, long delays and a challenging supply situation. Negotiations commenced, and kept managers, legal departments, patent attorneys, scientists and courier services busy for almost 2 years. Finally, over 30 interlocked legal documents were signed in 1997: all patent suits were dropped, and Waldheim (meanwhile, Sanochemia Pharmazeutika) became the exclusive supplier of synthetic galantamine for Janssen.

## Parallel & second-generation repurposing in neuropsychiatry

The history of repurposing attempts for galantamine did not end with the decision to develop it for Alzheimer’s disease, and went beyond studying the obvious use extensions for other types of dementia, mild cognitive impairment or cognitive impairment in schizophrenia and bipolar disorder, which are too numerous to be reviewed here. Instead, we shall focus on how other aspects of the cholinergic system were exploited with galantamine.

A German 24-week randomized, placebo-controlled trial in 149 recently detoxified alcohol-dependent individuals missed its primary end point of relapse prevention; however, a *post hoc* analysis showed that galantamine might have reduced alcohol consumption in those who relapsed [18], and that it reduced cigarette consumption in study participants by about 10% [19]. This study was based on a patent by LTS Lohmann Therapie Systeme (EP 449247, jointly held with Hefa-Frenon Arzneimittel; priority date 1990) and was conducted using the prototype of a transdermal patch developed by LTS (patent application WO/1994/16707). Independently, the University of Pennsylvania has sponsored a small open-label Phase II clinical trial with the marketed oral presentation of galantamine in smoking abstinence (clinicaltrials.gov, NCT01548638), apparently with inconclusive results.

The potential usefulness of galantamine in autism, for which the first pilot investigation had been published in 2002 [20], was investigated in a Phase III clinical trial (NCT00252603) for which no results are available. However, another randomized, double-blind autism trial that used galantamine as an augmenter for the antipsychotic, risperidone in 40 pediatric outpatients showed significantly greater improvement in the irritability (p = 0.017) and lethargy/social withdrawal (p = 0.005) subscales of the Aberrant Behavior Checklist-Community Scale relative to placebo [21]. Shire Pharmaceutical’s patent application WO/1999/007359, for attention deficit disorder, was not followed up.

Chronic poststroke aphasia is another interesting potential application that is indirectly related to cognition and has shown some promise in a clinical pilot study [22].

## And beyond

All these additional attempts stayed ‘on-target’ – in other words, they exploited the drug’s well-established cholinergic mechanism, inhibition of cholinesterases and allosteric modulation of nicotinic acetylcholine receptors. However, some that went beyond the boundaries of neuropsychiatry still carry potential for success.

Bulgarian researchers had reported effects of galantamine in the treatment of ‘psychogenic sexual asthenia’ in 1974 [23]; Ciba-Geigy (later merged into Novartis) claimed it for physiologic male erectile impotence with a 1991 priority date in US patent 5,177,070, presenting no substantial data but referring to oral doses identical to those that would later be approved for the treatment of Alzheimer’s disease. No actual studies have been reported.

In contrast, claims for chronic fatigue syndrome and fibromyalgia made in international patent application WO/1992/20327 were followed up by a Phase II clinical trial sponsored by Shire Pharmaceuticals, but failed to demonstrate efficacy [24].

Tardive dyskinesia, the major side effect of antipsychotic treatment, may have drug-induced damage to striatal cholinergic neurons as one of its components. In a randomized postmarketing study (NCT00164242) sponsored by Ortho-McNeil Neurologics (the US distributor of Reminyl/Razadyne), galantamine reduced mean total Abnormal Involuntary Movement Scale (AIMS) scores more than placebo, but this difference was not statistically significant (p = 0.08), although patients initially assigned to galantamine showed a modest rebound reversal of AIMS scores after switching to placebo [25].

The central cholinergic system is also intimately involved in the regulation of the sleep-wake cycle, which prompted Janssen Pharmaceutica to file WO/2005/016327 claiming galantamine for a broad variety of sleep disorders. Individual inventors from Germany and Sweden had claimed galantamine for sleep apnea and snoring before, in WO/1997/22339; upper airway muscles are also under cholinergic control.

In WO/2007/016793, the University of Montreal claimed galantamine as a neuroprotectant for retinal ganglion cells in glaucoma and age-related macular degeneration. A 2013 paper from this investigator group shows that the protection appears to be indirect, caused by stabilization of the retinal microvasculature and enhanced blood flow in experimental glaucoma [26]. This might have been preempted by a paper published by Italian researchers 50 years earlier [27].

Cholinesterase inhibitors with long-lasting but reversible binding characteristics can protect the active site of the enzymes against organophosphor poisons that permanently inactivate the enzyme by covalent modification of amino acid residues. They can also be useful after exposure to these chemical warfare agents and agricultural insecticides because they partially prevent inactivation of the newly synthesized enzymes by residual poison after decontamination. WO/2006/036686 and WO/2010/025259 were filed by the University of Maryland to secure claims to that effect. In guinea pigs, galantamine is effective even after exposure to lethal doses of the irreversible high-affinity cholinesterase inhibitor *O*-ethyl-*S*-(2-diisopropylaminoethyl) methylphosphonothiolate, better known as nerve gas VX, which overwhelms several established antidotes [28].

Finally, galantamine can significantly influence the immune response in tularemia *via* the cholinergic anti-inflammatory pathway by upregulating IFN-γ and downregulating IL-6, as was shown in mice experimentally infected with *Francisella tularensis* [29].

As all other cholinergic drugs that are approved for Alzheimer’s disease, galantamine has a distinct side effect profile that must enter the equation, which determines the acceptable balancing point between safety and efficacy for every therapeutic use. This tipping point is already in a problematic territory, if cholinesterase inhibitors are used in mild cognitive impairment [30], and galantamine would certainly not be appropriate for minor disorders unless systemic exposure is significantly lower than with Alzheimer’s disease.

## The take-home message

The story of galantamine is a prototypical case of simple, rational and purely mechanism-based drug repurposing: if the mechanism of a drug is known, one can look at additional medical conditions that might benefit from this mechanism. Galantamine affects the cholinergic system, which leaves few mammalian functions untouched, and it does so to the exclusion of almost every other direct target. As an orally bioavailable brain-penetrating agent that has a reversible action in the range of hours, a high volume of distribution in the body, and is uncomplicated in terms of metabolism and excretion, it is bound to offer many therapeutic possibilities.

What this case illustrates is that not all of these theoretical opportunities translate into clinical success; with a repurposing project, clinical studies can fail (and can fail for no apparent reason) in the same way as with a new chemical entity. Of equal importance, a compound that shines in its clinical repurposing program can face additional hurdles that are not related to the medical and pharmacological sciences. Pronounced problems with reliable sourcing at acceptable and predictable prices, as well as intellectual property issues, might prove challenging even for top-ten pharma companies; but even small entities can achieve considerable leverage if they play their cards well. Drug repurposing is a multidisciplinary business where sometimes many strings need to be pulled with the correct force, and in the correct sequence.

## Future perspective

Galantamine is a prime example of drug repurposing, an approach to drug development which aims at more comprehensive therapeutic exploitation of known molecules. The next 5–10 years will likely show that a large number of compounds, discontinued drug candidates as well as established drugs, such as galantamine, which no longer have patent exclusivity under their original uses, can be redeveloped with reduced risk, based on existing human use data. As our knowledge of biological pathways and their complex interactions improves, such opportunities can be investigated with increased confidence.

**Executive summary**

Galantamine was developed as a centrally acting and neuromuscular drug in the 1960s, exploiting its cholinergic activity.
Positive effects on artificially impaired human cognition were reported in 1977, but systematic redevelopment as a drug for Alzheimer’s disease did not commence until the early 1990s.
Restricted availability from plant sources and a fragmented intellectual property situation offered considerable obstacles to development.
The potential of galantamine is not nearly exhausted, as many attempts at drug repurposing illustrate. Although some suggested applications appear nonobvious, they all rely on the drug’s cholinergic activity.
